# Global burden and epidemiological trends of paediatric-onset multiple sclerosis: a cross-sectional analysis of the 2021 global burden of disease study

**DOI:** 10.3389/fped.2025.1679340

**Published:** 2025-12-15

**Authors:** Ziliang Zhuo, Zongbo Zhao, Manyun Yan, Jianing Tan, Yang Gu, Wei Zhong, Yao Zhang, Hui Liu

**Affiliations:** 1 Department of Neurology, Affiliated Changshu Hospital of Nantong University, Changshu, China; 2 Department of Neurology, Pukou Hospital of Chinese Medicine Affiliated to China Pharmaceutical University, Pukou, China

**Keywords:** multiple sclerosis, global burden, epidemiology, pediatric, paediatric-onset multiple sclerosis

## Abstract

**Background:**

Paediatric-onset Multiple Sclerosis (POMS) is a rare autoimmune disorder of the central nervous system, primarily affecting children and adolescents, characterized by recurrent episodes of neurological impairment. This study delineates the global disease burden and epidemiological characteristics of this condition.

**Methods:**

Data on POMS were obtained from the 2021 Global Burden of Disease (GBD) Study, including estimates from 1990 to 2021. We described the global burden of POMS using incidence, prevalence, DALYs (Disability-Adjusted Life Years), YLDs (Years Lived with Disability), and age-standardized rates. We utilized the Joinpoint Regression Program to analyze the epidemiological trends of POMS disease burden over the past three decades. The Nordpred model was employed to project the future disease burden of POMS.

**Results:**

In 2021, the global incidence number of POMS was 1,899 (95% CI: 1,096–2,868), with an ASIR of 0.09 (95% CI: 0.05–0.14). The global prevalence number of POMS was 5,276 (95% CI: 2,921–7,880), with an ASPR of 0.25–95% CI: 0.14−0.38). The global DALYs for POMS was 3,073 (95% CI: 2,216–4,142), with an age-standardized DALYs rate of 0.15 (95% CI: 0.10–0.20). The global YLDs for POMS was 1,489 (95% CI: 737–2,529), with an age-standardized YLDs rate of 0.07 (95% CI: 0.03–0.12). Using Joinpoint Regression, we found that during 1990–2021, the AAPC for global ASIR was 0.07 (95% CI: 0.06–0.07), for global ASPR was 0.05 (95% CI: 0.05–0.05), for global age-standardized DALYs rate was −0.31 (95% CI: −0.34 to −0.28), and for global age-standardized YLDs rate was 0.05 (95% CI: 0.05–0.05). According to the Norpred model, by 2046, the global incidence number of POMS is projected to be 1,760 with an ASIR of 0.14. The global prevalence number of POMS is projected to be 4,915 with an ASPR of 0.38. The global DALYs number of POMS is projected to be 2,694 with an age-standardized DALYs rate of 0.21. The global YLDs number of POMS is projected to be 1,387 with an age-standardized YLDs rate of 0.11.

**Conclusion:**

POMS is characterized by a relatively low disease burden; however, it has shown a persistent upward trend in recent years. It is necessary to increase focus on this disease and develop new therapeutic approaches.

## Introduction

Multiple sclerosis (MS) is a chronic inflammatory demyelinating disorder of the central nervous system (CNS) that primarily affects myelin sheaths of nerve fibers in regions including the brain and spinal cord (1). The etiology of MS is incompletely understood but may involve viral infections, autoimmune reactions, genetic factors, and environmental triggers (2). MS can develop at any age but shows higher prevalence in young adults (3). While characterized by relatively low mortality, it frequently relapses and necessitates long-term management (4). Importantly, MS can also occur in children (5). Pediatric patients typically experience acute onset (6). Initial symptoms include visual decline, diplopia, or ophthalmoplegia; mono- or poly-limb paralysis; sensory abnormalities; ataxia; urinary/bowel dysfunction; cognitive or affective alterations (7). Symptoms such as fever, headache, nausea, vomiting, or seizures can occur in some patients with POMS during their first episode (8). The typical disease course of POMS involves alternating relapses and remissions (9). The average age at first diagnosis of POMS is 12.3 years, and it leads to a significant disease burden (10).

Understanding the epidemiological trends and disease burden of POMS facilitates the development of targeted prevention and control measures. The epidemiology of POMS involves consideration of genetic and environmental factors, with its burden varying significantly across geographical locations and ethnic groups (11). Differential diagnosis of multiple sclerosis may pose particular challenges for patients from Latin America, Africa, the Middle East, Eastern Europe, Southeast Asia, and the Western Pacific, where environmental factors, genetic backgrounds, and healthcare accessibility differ considerably from North America and Western Europe—regions with the highest MS prevalence (12). The Global Burden of Disease (GBD) database, as the world's most comprehensive health repository, collates burden data for 459 diseases and injuries across 204 countries. Previous studies have utilized GBD 2016 data to assess the disease burden of MS in the general population (13). However, reports on the disease burden and epidemiological trends of POMS remain limited. Using GBD 2021 data, we report the incidence, prevalence, Disability-Adjusted Life Years (DALYs), Years Lived with Disability (YLDs) for POMS and evaluate its epidemiological trends. We aim to conduct a secondary analysis of data related to the disease burden of POMS to thoroughly document its burden and epidemiological trends, thereby providing a reference for the development of targeted prevention and control strategies.

## Methods

### Data sources

The Global Burden of Disease (GBD) 2021, accessible at: https://vizhub.healthdata.org/gbd-results/, constitutes a comprehensive analysis that aggregates diverse data from sources including surveys, censuses, vital registration systems, and health records. These data enable the estimation of key health metrics such as incidence, prevalence (14). GBD employs the International Classification of Diseases (ICD) system for disease coding and classification, ensuring standardized global health trend comparisons. The Global Health Data Exchange (GHDx, available at: https://www.healthdata.org/) provides an interactive interface for accessing this extensive repository, supporting data exploration by researchers and policymakers. For this study, complete datasets on incidence, prevalence, Disability-Adjusted Life Years (DALYs) and Years Lived with Disability (YLDs) related to POMS were obtained from GBD 2021. This dataset covers ages 0–≥95 years during the period 1990–2021, establishing a robust analytical foundation (15).

The GBD study synthesizes a wide range of data sources, including vital registration systems, population health surveys, hospital records, and peer-reviewed studies retrieved from PubMed/Embase. It encompasses individuals of all ages, from newborns to adults over 95 years old, and both sexes, making it a crucial resource for global health research. The GBD database applies the following exclusion criteria: sources with poor data quality, unclear methodological descriptions, or significant bias are excluded; data incompatible with the core GBD case definitions are also excluded. For partially missing data across years or regions, GBD utilizes advanced statistical modeling techniques, including spatiotemporal Gaussian process regression, to generate estimates (16).

Based on the “Cause of death or injury” category in the GBD 2021 database, we obtained the disease burden of multiple sclerosis. Then we extracted the number of incidence, prevalence cases and DALYs, YLDs years of multiple sclerosis for 4 age groups: “<5”, “5 to 9”, “10 to 14”, “0 to 14”.

To obtain the disease burden in the children, we used the Age-standardized rate (per 100,000 population). Age-Standardized Rate was calculated to enable comparisons across regions and time periods by accounting for changes in population age structure. The absolute number of incident cases was also reported to quantify real-world public health burden, particularly to inform resource allocation. The data on age-standardized rates include the Age-standardized incidence rate (ASIR), Age-standardized prevalence rate (ASPR), Age-standardized DALYs rate, and Age-standardized YLDs rate. Age standardization of the data was performed using the direct method, which allows for the comparison of disease burden by removing the confounding effect of differences in age distribution (17). This calculation was executed using the ageadjust.directfunction from the epitoolspackage. The function requires three inputs: the number of observed cases and the population size in each age group within the study population, along with the proportional age distribution of a standard population. This process yields age-standardized estimates, enabling comparable assessments of disease burden across different time periods and regions and providing a more accurate reflection of epidemiological distribution characteristics (17).

### Average annual percent change

The Joinpoint Regression Program (Version 5.1.0.0) was used to analyze trends in the age-standardized rate of POMS from 1990 to 2021, generating average annual percent changes (AAPC) with corresponding 95% confidence intervals (CI). Joinpoint is specialized statistical software employing joinpoint regression modeling for trend analysis. It processes time-series data to identify the most parsimonious joinpoint model supported by the data, allowing users to specify minimum and maximum joinpoints for evaluation (18).

### Prediction of future disease burden

Building upon the Norpred prediction framework, we conducted a systematic forecast of the future disease burden of POMS. The Norpred model is a predictive tool originally developed for cancer incidence and mortality based on the age-period-cohort modeling framework (19). This approach constructs generalized linear models that incorporate both period effects and birth cohort effects to linearly extrapolate future incidence and mortality trends. The model operates under the core assumption that disease rates are influenced not only by individual age but also by temporal period effects and birth cohort characteristics. Previous studies have utilized this model to predict regional disease burden of MS (20).

## Results

### Global burden analysis

**Figure 1:**
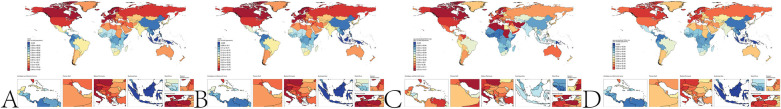
The age-standardized rate of POMS from 204 countries and territories in 2021. **(A)** Age-standardized incidence rate (ASIR) of POMS. **(B)** Age-standardized prevalence rate (ASPR) of POMS.**(C)** Age-standardized DALYs rate of POMS. **(D)** Age-standardized YLDs rate of POMS.

**Figure 2:**
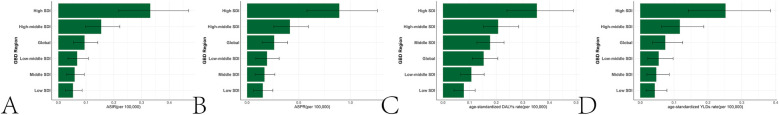
The age-standardized rate of POMS across high-low SDI regions in 2021. **(A)** ASIR of POMS. **(B)** ASPR of POMS. **(C)** Age-standardized DALYs rate of POMS. **(D)** Age-standardized YLDs rate of POMS.

**Figure 3:**
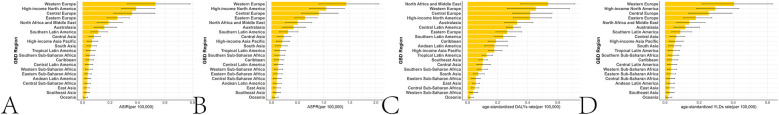
The age-standardized rate of POMS across 21 super SDI regions in 2021. **(A)** ASIR of POMS. **(B)** ASPR of POMS. **(C)** Age-standardized DALYs rate of POMS. **(D)** Age-standardized YLDs rate of POMS.

**Figure 4:**
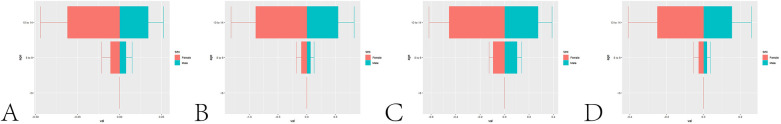
The age-standardized rate of POMS across age groups in 2021. **(A)** ASIR of POMS. **(B)** ASPR of POMS. **(C)** Age-standardized DALYs rate of POMS. **(D)** Age-standardized YLDs rate of POMS.

In 2021, the global incidence number of POMS was 1,899 (95% CI: 1,096–2,868), with an ASIR of 0.09 (95% CI: 0.05–0.14). The global prevalence number of POMS was 5,276 (95% CI: 2,921–7,880), with an ASPR of 0.25 (95% CI: 0.14–0.38). The global DALYs for POMS was 3,073 (95% CI: 2,216–4,142), with an age-standardized DALYs rate of 0.15 (95% CI: 0.10–0.20). The global YLDs for POMS was 1,489 (95% CI: 737–2,529), with an age-standardized YLDs rate of 0.07（95% CI: 0.03–0.12). The global burden of POMS across countries and territories in 1990 and 2021 is detailed in Supplementary Tables S1–S4 in Appendix. Figure 1 illustrates the 2021 global disease burden of POMS by region, and country. The age-standardized burden of different types of POMS in different SDI regions is shown in Figures 2 and 3. The age-standardized burden of different age groups of POMS is shown in Figure 4.

### Temporal trends in the epidemiology of POMS, 1990–2021

**Figure 5:**
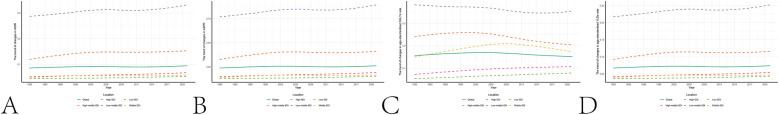
Temporal trends in POMS across global and SDI regions. **(A)** Temporal trends of ASIR of POMS. **(B)** Temporal trends of ASPR of POMS.**(C)** Temporal trends of age-standardized DALYs rate of POMS. **(D)** Temporal trends of age-standardized YLDs rate of POMS.

Using Joinpoint Regression, we found that during 1990–2021, the AAPC for global ASIR was 0.07(95% CI: 0.06–0.07), for global ASPR was 0.05 (95% CI: 0.05–0.05), for global age-standardized DALYs rate was −0.31 (95% CI: −0.34 to −0.28), and for global age-standardized YLDs rate was 0.05 (95% CI: 0.05–0.05). Figure 5 and Supplementary Table S5 in Appendix shows the trends in POMS prevalence globally and across SDI regions.

### Prediction of POMS

**Figure 6:**
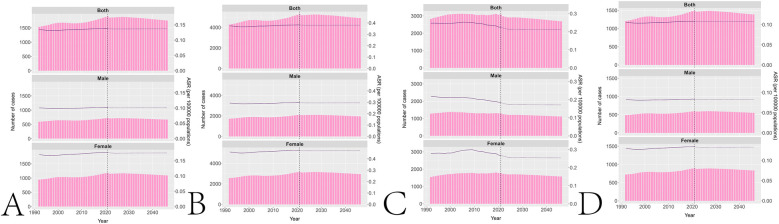
Prediction of POMS. **(A)** Prediction of ASIR. **(B)** Prediction of ASPR. **(C)** Prediction of age-standardized DALYs rate. **(D)** Prediction of age-standardized YLDs rate.

According to the Norpred model, by 2046, the global incidence number of POMS is projected to be 1,760 with an ASIR of 0.14. The global prevalence number of POMS is projected to be 4,915 with an ASPR of 0.38. The global DALYs number of POMS is projected to be 2,694 with an age-standardized DALYs rate of 0.21. The global YLDs number of POMS is projected to be 1,387 with an age-standardized YLDs rate of 0.11. Figure 6 and Supplementary Tables S6–S9 in Appendix illustrates the projected disease burden of POMS by 2046 based on the Norpred model.

## Discussion

This study presents the first global quantification of the disease burden of POMS. Leveraging the GBD2021 database, our findings reveal that in 2021, the global incident cases of POMS approached 2,000. According to data from the GBD 2021 study, there were 1,899 new cases of POMS globally in 2021. For context, the total number of new MS cases worldwide reported for 2021 was 62,920. This indicates that the incidence of POMS is lower than that of adult-onset MS (21). Similar to the pattern observed in adult MS, the distribution of new POMS cases is uneven across regions with different SDI levels. Specifically, high-SDI regions reported a greater number of new POMS cases compared to low-SDI region.

The global barriers to the diagnosis of Multiple Sclerosis warrant further attention (22). The new 2024 McDonald Criteria address how to achieve an early diagnosis of MS and strive to make diagnostics more accessible for people worldwide, including those with POMS (23). The most immediate benefit of the 2024 McDonald Criteria is the significant reduction in the time from the first appearance of symptoms to a confirmed diagnosis (24). This secures a critical window for early initiation of high-efficacy disease-modifying therapies, thereby enabling better disease control and improving long-term patient outcomes (25). These criteria provide specific considerations for children, the elderly, and individuals with comorbidities (26). The revision process took into account regions with high, medium, and low resources, aiming for global uniform application to reduce diagnostic delays caused by resource disparities (27). It is noteworthy that Adult-Onset Multiple Sclerosis (AOMS) and POMS are essentially the same disease manifesting at different ages. For individuals with POMS, proactive early and high-efficacy interventions, akin to those used for adult MS, are crucial (28).

POMS frequently manifests with non-specific initial symptoms, including headache and fever in numerous pediatric patients (29). The disease typically exhibits acute onset and is primarily diagnosed using the McDonald criteria (30). Radiologists must possess a profound understanding of diverse demyelinating disorders that may occur during childhood to ensure accurate diagnosis and facilitate early therapeutic intervention (31).

Approximately 5,000 individuals worldwide are currently living with POMS. Novel therapeutic approaches for POMS warrant increased attention (32). However, compared to adult treatments, the development, testing, and regulatory approval of POMS therapies progress significantly slower and face ethical constraints (8). Despite these challenges, pediatric trials of drugs approved for adult-onset multiple sclerosis are emerging (33). Early intervention is recognized as critical for improving long-term outcomes in POMS (34).

Joinpoint regression analysis revealed positive AAPC values for global ASIR, ASPR, and age-standardized YLDs rate in POMS, indicating a progressive increase in disease burden despite its low-prevalence status. Notably, the age-standardized DALYs rate demonstrated a declining trend. Recent advancements in MRI accessibility and updated diagnostic criteria have facilitated the identification of previously undetected POMS cases (35). Concurrently, therapeutic innovations like anti-CD20 monoclonal antibodies have contributed to reduced DALYs (36). Critically, however, POMS-induced neurological damage remains irreversible, underscoring the paramount importance of early diagnosis and prompt therapeutic intervention (37). The Norpred model projects that by 2046, POMS would continue to exhibit a distinct epidemiological profile characterized by rising incidence, prolonged survival, and increased disability burden. Enhancing early diagnostic capabilities and developing novel therapeutic approaches for POMS remain critical priorities. Integration of AI-assisted diagnostic methods into clinical workflows should be actively considered (38). Similarly, radiomics technology shows potential for predicting the onset of POMS (39). Improved diagnostics, changes in coding practices, and survival bias may also exert an influence on the results of Joinpoint regression analysis.

This study has several limitations that warrant careful consideration. First, and most critically, the GBD estimates are model-dependent and may be particularly fragile for a rare disease such as POMS, especially in low- and middle-income countries where primary epidemiological data are scarce or non-existent. In these regions, estimates are largely imputed through statistical modeling rather than based on robust observed data, which significantly undermines the reliability of cross-country comparisons. The wide confidence intervals (e.g., prevalence 2,921–7,880) we report directly reflect this high uncertainty. Second, consistent with the inherent data challenges, the Norpred model shows widening confidence intervals with longer projection periods, further reducing forecast reliability. MS is known to coexist with several autoimmune diseases, including autoimmune thyroid diseases, inflammatory bowel disease, and psoriasis (40). Smoking is also recognized as a risk factor for MS in the GBD database. However, there is currently no reported research on which specific diseases coexist with POMS. For younger age groups, attributable risk factor data for MS are very limited or non-existent. This is likely because MS is relatively rare in children and adolescents, leading to insufficient population-based epidemiological data. Consequently, the GBD model cannot generate statistically robust risk estimates for these age groups, highlighting a significant gap that future research needs to address.

## Conclusion

POMS is characterized by a relatively low disease burden; however, it has shown a persistent upward trend in recent years. It is necessary to increase focus on this disease and develop new therapeutic approaches.

## Data Availability

Publicly available datasets were analyzed in this study. This data can be found here: Visit the Global HealthData Exchange GBD 2021 data-input sources tool at https://vizhub.healthdata.org/gbd-results/.
